# Maxillary Odontogenic Keratocyst

**DOI:** 10.1093/jscr/rjac078

**Published:** 2022-04-11

**Authors:** Michael Walsh, Mansoor A Hussein, Marguerite Carter, Shawkat Abdulrahman

**Affiliations:** Department of Otorhinolaryngology, Tallaght University Hospital, Dublin, Ireland; Department of Otorhinolaryngology, Tallaght University Hospital, Dublin, Ireland; Pathology Department, Tallaght University Hospital, Dublin, Ireland; Department of Otorhinolaryngology, Tallaght University Hospital, Dublin, Ireland

**Keywords:** Odontogenic Keratocyst, maxillary sinus, endoscopic sinus surgery, Carnoy’s solution

## Abstract

The Odontogenic Keratocyst (OKC) is one of the most aggressive odontogenic cysts. OKCs of the maxilla are particularly rare with less than 1% of cases reported in the literature. A 29-year-old female patient presented with pain and loose upper molars. Imaging confirmed an ectopic tooth at the osteomeatal complex and a maxillary OKC. These were endoscopically surgically removed and two teeth were encountered at the maxillary antrum. Histopathology confirmed the diagnosis of OKC of the maxilla. Surveillance with CT imaging and clinical assessment at 6 months shows no evidence of recurrence.

## INTRODUCTION

The Odontogenic Keratocyst (OKC) is one of the most aggressive odontogenic cysts owing to its high recurrence rate and tendency to invade adjacent tissues [1]. OKC’s arise from the remnants of the dental lamina [2] and can occur in isolation or as a multitude of cysts which are linked to syndromes such as Gorlin–Galtz. OKC’s may be regarded as a benign neoplasm rather than a conventional cyst [3]. The World Health Organization (WHO) however reclassified the OKC as Keratocystic odontogenic tumour (KCOT) in 2005. As of 2017, the WHO again switched KCOT back to OKC as there was a lack of support, for their justification as a tumour entity [4].

OKCs of the maxilla are particularly rare with less than 1% of cases reported in the literature [5]. The case presented outlines the management and findings of a solitary OKC in the maxilla and why multidisciplinary team input is important in diagnosing rare pathology and achieving best outcomes.

## CASE REPORT

**Figure 1:**
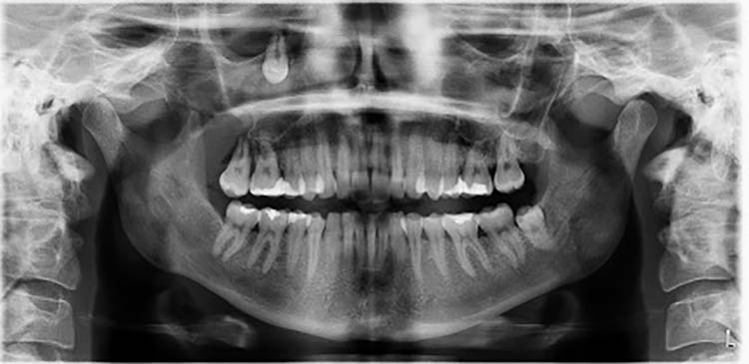
Orthopantomogram depicting the ectopic tooth in the right maxillary sinus.

**Figure 2:**
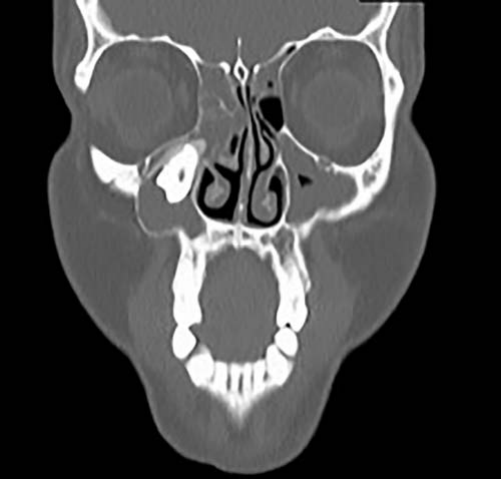
Coronal CT image of the right ectopic tooth at the antrum of the right maxillary sinus.

**Figure 3:**
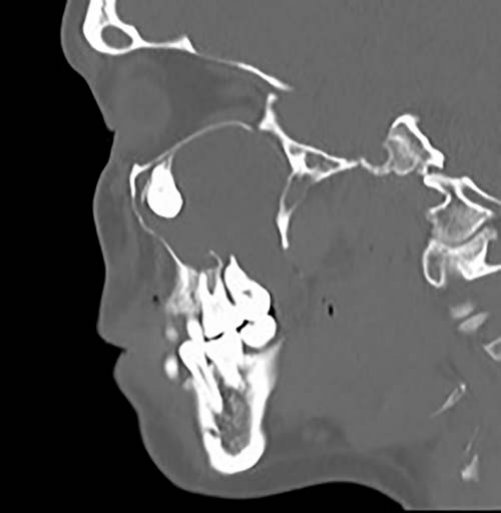
Sagittal CT image of the right ectopic tooth at the antrum of the right maxillary sinus.

A 29-year-old female patient was referred from the Maxillofacial surgeons to ENT outpatients, for assessment of a loose upper right molar and right-sided retro-orbital pain, ongoing for 4 months. She had no past medical or surgical history and was a lifelong non-smoker with no significant family history. Imaging with a orthopantomogram (Fig. 1) depicted an ectopic tooth in the right maxillary sinus. CT imaging further characterized a thin-walled cystic mass in the right maxillary sinus (Figs 2 and 3), and opacification of the maxillary and ethmoidal air cells is also visualized alongside the afore mentioned ectopic tooth.

**Figure 4:**
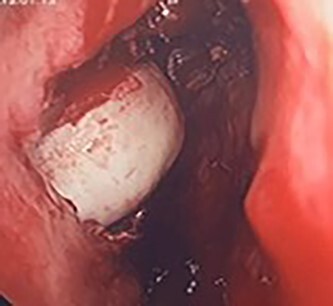
Intra-operative image of the ectopic tooth at the right osteomeatal complex.

**Figure 5:**
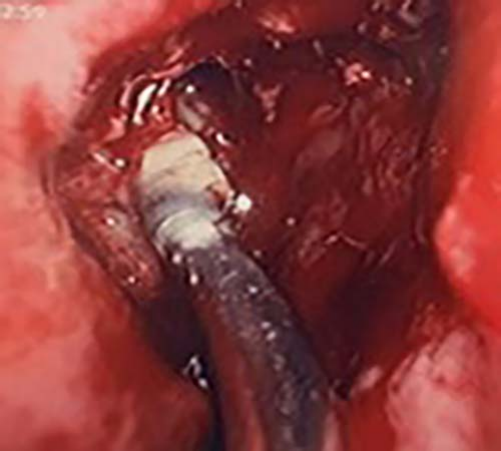
Intra-operative image of the ectopic tooth at the right osteomeatal complex, with curved suction facilitating enucleation.

**Figure 6:**
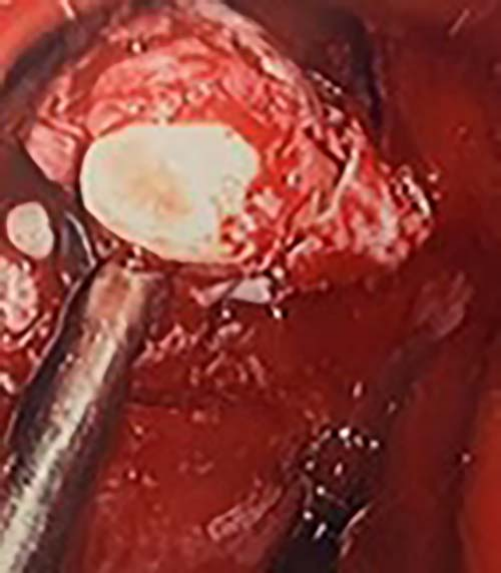
Intra-operative image of the ectopic tooth at the right osteomeatal complex, with curved suction facilitating enucleation.

**Figure 7:**
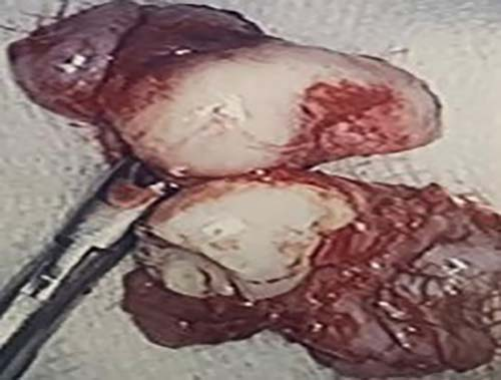
Two extracorporeal teeth that were removed from the right maxillary sinus.

**Figure 8:**
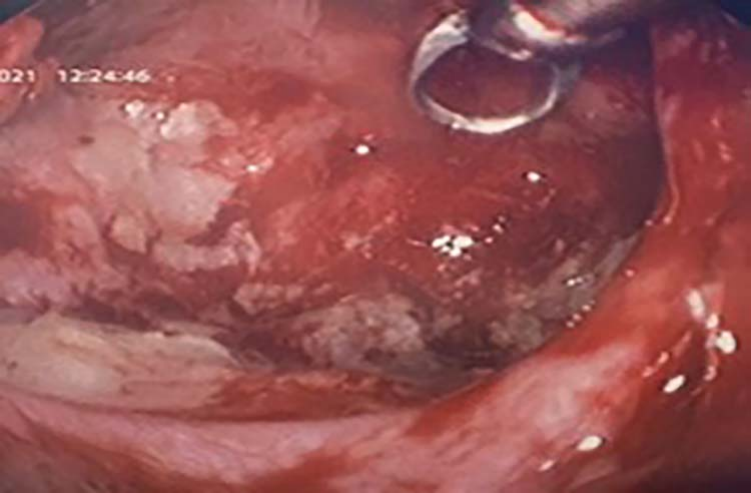
Intra-operative image with a 70° scope depicting the cleared maxillary sinus cavity, the scope facilitated cautery at the base of the cyst.

Flexible nasendoscopy offered little in terms of diagnostic benefit. An exam under anesthetic with a view to removing the ectopic tooth via endoscopic sinus surgery was warranted. Right middle turbinate trimming along with a right maxillary antrostomy was performed. The tooth was immediately identified at the antrum as depicted in Figs 4–6. The bony capsule of the tooth was entered and, on manipulation, a second ectopic tooth was identified (Fig. 7) both of which were removed en bloc. The cystic component within the maxillary sinus was marsupilized and extracted. The sinus cavity can be viewed with the utilization of 70° endoscope (Fig. 8). This facilitated bipolar cautery of the base of the cyst and confirmed the absence of an oroantral fistula.

**Figure 9:**
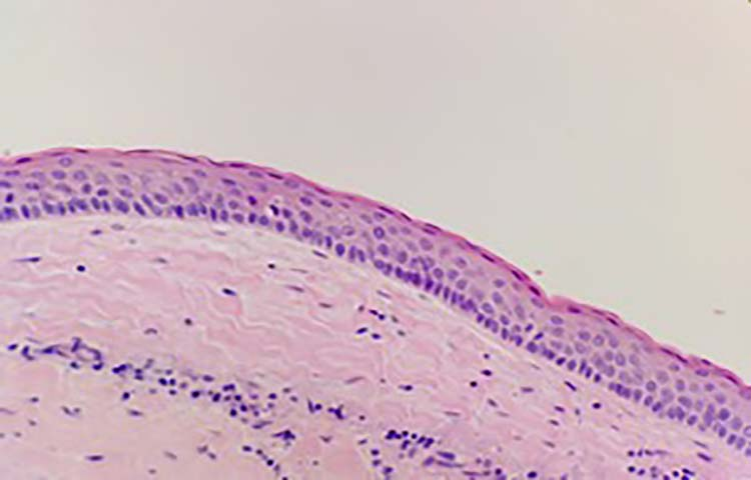
Keratinizing squamous epithelium with prominent basal palisading (H&E stain, 400× magnification).

Histological assessment confirmed parakeratinized epithelium with a prominent palisaded basal layer. The findings were confirmatory for an OKC (Fig. 9). Clinical review at 6 months depicted no evidence of recurrence. A CT for further assessment is planned, with consideration of an examination under anesthetic, with the potential for pre-lacrimal stent insertion to facilitate Sino-antral washout.

## DISCUSSION

OKCs of the maxilla are atypical, and they are more often found in the canine region. The findings on CT imaging located the cyst base at the second upper molar, and this would be more representative of a radicular cyst. The ectopic eruption of teeth in regions other than the oral cavity is rare [6], though they have been reported. However, to find a tooth at the osteomeatal complex is extremely atypical, there is postulation that with the development of an OKC, it encroaches on the space of the sinus and displaces its borders, displacing teeth buds and resulting in an eruption of an ectopic tooth [7].

Histologically, the subtypes of OKC have been divided into parakeratinized and orthokeratotic. The parakeratotic subtype is the most frequent (80%) and is significantly more aggressive than its counterpart, with a high propensity for recurrence [8]. Those associated with Gorlin–Galtz syndrome are likely to have multiple cysts, dental, skeletal, neurological and ophthalmic abnormalities and a higher rate of recurrence. Our patient did not exhibit any of said features and did not warrant further investigation.

Given the concern regarding recurrence, Carnoy’s solution had been proposed as a adjuvant treatment post-marsupialization. The solution itself is made up of chloroform, absolute ethanol, glacial acetic acid and ferric chloride. A systematic review of Carnoy’s solution used in treating OKC’s categorized the evidence as grade C [9]. There is a risk regarding damage to soft tissue and neurovascular structures with its application, and for this reason, it was not utilized in this circumstance. Should the OKC recur utilization of Carnoy’s solution may be employed to minimize future surgical intervention. Given the patients age and low chance of malignant transformation, the more extensive surgical option of a maxillectomy and flap reconstruction was not indicated.

There is evidently the need for multidisciplinary team input and good communication amongst specialties when dealing with rare pathology to achieve correct diagnosis and best patient outcomes. This patient will require lifelong surveillance given the propensity for recurrence. The role for adjuvant treatment is still available should the need arise.

## CONFLICT OF INTEREST STATEMENT

The authors do not declare any conflict of interest.
